# Endoscopic and Histopathological Findings of the Esophagus, Stomach, and Duodenum in Patients with Crohn’s Disease from a Reference Center in Bahia, Brazil

**DOI:** 10.3390/clinpract11020052

**Published:** 2021-06-15

**Authors:** Andrea Maia Pimentel, Luiz Antônio Rodrigues de Freitas, Rita de Cássia Reis Cruz, Isaac Neri de Novais Silva, Laíla Damasceno Andrade, Paola Nascimento Marques, Júlia Cordeiro Braga, Flora Maria Lorenzo Fortes, Katia Rejane Marques Brito, Jaciane Araújo Mota Fontes, Neogélia Pereira Almeida, Valdiana Cristina Surlo, Raquel Rocha, André Castro Lyra, Genoile Oliveira Santana

**Affiliations:** 1 Medicine and Health Science Postgraduate Program, Federal University of Bahia, Salvador 40026-010, Brazil; deamaiap@hotmail.com (A.M.P.); alyra@live.com (A.C.L.); 2 Pathology Laboratory, Gonçalo Moniz Institute Fiocruz Bahia, Salvador 40296-710, Brazil; lfreitas@bahia.fiocruz.br; 3 Gastroenterology Unit, Hospital Geral Roberto Santos, Salvador 45675-000, Brazil; ritapedro2004@yahoo.com.br (R.d.C.R.C.); florafortes@yahoo.com.br (F.M.L.F.); katiabritomed@hotmail.com (K.R.M.B.); jacimota100@hotmail.com (J.A.M.F.); neoalmeida@yahoo.com.br (N.P.A.); surlomed@hotmail.com (V.C.S.); 4 Department of Life Sciences, Universidade do Estado da Bahia, Salvador 41195-001, Brazil; isaacneri21@gmail.com (I.N.d.N.S.); pnascimentomarques@gmail.com (P.N.M.); jcbrbraga@gmail.com (J.C.B.); 5 Medical School, Centro Universitário UniFTC, Salvador 41741-590, Brazil; laiandrade@outlook.com; 6 Department of Sciences of Nutrition, School of Nutrition, Federal University of Bahia, Salvador 41195-00, Brazil; raquelrocha2@yahoo.com.br

**Keywords:** Crohn’s disease, gastritis, enhanced focally gastritis, granuloma, *Helicobacter pylori*

## Abstract

(1) The aim of the present study was to describe the endoscopic and histopathological findings in the esophagus, stomach, and duodenum in patients with Crohn’s disease. (2) Methods: This was a cross-sectional study that included patients receiving treatment from the inflammatory bowel disease outpatient clinic. Esophagogastroduodenoscopies with biopsies of the stomach and proximal duodenum were performed. Presence of *Helicobacter pylori* bacteria was assessed by Giemsa staining. (3) Results: We included 58 patients. Erosive esophagitis was identified in 25 patients (43.1%), gastritis was diagnosed in 32 patients (55.2%) and erosive duodenitis was found in eight (13.8%). The most frequent histopathological finding in the *H. pylori*-positive group was increased inflammatory activity in the gastric body and antrum, with a predominance of mononuclear and polymorphonuclear cells. In turn, the most frequent finding in the *H. pylori*-negative group was chronic inflammation with predominance of mononuclear cells. Focally enhanced gastritis was identified in four patients (6.9%), all of whom were negative for *H. pylori*. Granulomas were not observed. *H. pylori* infection was present in 19 patients (32.8%). (4) Conclusions: Nonspecific endoscopic and histological findings were frequent in patients with Crohn’s disease. Focally enhanced gastritis was uncommon and observed only in *H. pylori-*negative patients. The time from the diagnosis, patient age, and therapy in use may have influenced the nondetection of epithelioid granuloma.

## 1. Introduction

The prevalence of upper gastrointestinal (GI) tract involvement in symptomatic patients with Crohn’s disease (CD) varies between 0.5 and 5% [1,2,3]. More recent observational studies, in which esophagogastroduodenoscopy (EGD) was performed as a routine part of the diagnostic evaluation, have demonstrated a higher frequency of both endoscopic and histopathological findings [4,5].

There is no recommendation to perform EGD in asymptomatic adult patients with CD. Studies performed in the pediatric population have demonstrated that the involvement of the upper GI tract has a higher prevalence; it is thus recommended to perform EGD with biopsies of the upper gastrointestinal tract routinely at the time of diagnosis in pediatric patients [6,7]. 

Similarly to the involvement of the lower digestive tract, endoscopic findings are not specific and include erosions, aphthous ulcers, longitudinal ulcers, strictures, and fistulas [8]. The main histological findings described are chronic inflammation, lymphoid aggregates, fibrosis, focally enhanced gastritis (FEG), and epithelioid granulomas [9,10]. The lower prevalence of *H. pylori* infection in patients with CD is a topic that is still debated, with studies showing conflicting results [11,12].

In the non-Caucasian population, these alterations are studied less often [13]. The aim of the present study was to describe the main endoscopic and histopathological findings of the esophagus, stomach, and duodenum in a sample of patients with CD being treated in a reference outpatient clinic for IBD (inflammatory bowel disease) in Brazil.

## 2. Materials and Methods

### 2.1. Study Design and Subjects

This was an observational, cross-sectional study that included patients with CD, aged 18 years or older, from June 2015 to April 2018. These patients were under treatment in one public reference unit for patients with IBD.

The CD diagnoses were based on clinical, laboratory, endoscopic, radiological, and histopathological findings, according to the ECCO (European Crohn’s and Colitis Organisation) guidelines [6,14]. Participants answered a questionnaire regarding clinical and demographic data. The Montreal classification [15] was applied and the Harvey Bradshaw Index (HBI) [16] was used for evaluation of disease activity. We considered any alcohol intake or cigarette use to be active alcohol consumption and active smoking. The presence of anemia and C-reactive protein (CRP) values were collected in medical records. Patients were instructed to discontinue the use of proton pump inhibitors (PPIs) for at least seven days and of nonhormonal anti-inflammatory drugs (NSAIDs) for at least 30 days prior to EGD, if they were being used. Exclusion criteria were as follows: uncompensated comorbidities and the use of anticoagulants or coagulopathies, which would be contraindications to performing EGD with biopsies.

### 2.2. Esophagogastroduodenoscopy

The participants were submitted to EGD conducted by a single endoscopist (RCRC), who was blinded to the clinical and laboratory data. According to the protocol, two biopsies were performed of the body, antrum, and proximal portion of the duodenum (bulb and second portion) during the procedure. In addition, biopsies were obtained of any endoscopically altered areas. The Los Angeles [17], Sydney [18], and Sakita classifications were used for gastroesophageal reflux disease, gastritis, and ulcers, respectively.

### 2.3. Histopathology

Histopathological analyses were performed by a single pathologist (LARF), who was blinded to the clinical, laboratory, and endoscopic patient data. Biopsy samples were fixed in 10% buffered formalin and stained with hematoxylin and eosin. Giemsa staining was used to identify *H. pylori* bacteria.

### 2.4. Statistical Analysis

Categorical variables were described as absolute frequencies and percentages. Continuous variables were described as the mean ± standard deviation or median and range. The associations between categorical variables were assessed using the chi-squared test or Fisher’s exact test. The The statistical analysis was performed using SPSS software version 21.0 (SPSS Inc., Chicago, IL, USA). A *p*-value of less than 0.05 was considered statistically significant. 

### 2.5. Ethical Considerations

The study was designed according to ethical and bioethical considerations, as well as the ethical guidelines of the 1975 Declaration of Helsinki and according to current norms. It was submitted and approved by the Research Ethics Committee of the Roberto Santos General Hospital, under number 1.115.522. The patients agreed to participate in the study and signed a free informed consent form before application of the questionnaire, review of the chart, and being submitted to the procedure (EGD with biopsies and histopathological study).

## 3. Results

### 3.1. Demographic and Clinical Characteristics

The demographic and clinical characteristics of the subjects included are listed in Table 1.

The mean age at the time of EGD was 42.1 (±12.8). The median time of diagnosis was 48 months (1-312). In relation to ethnicity, 10 patients (17.2%) declared themselves as white, 16 patients (27.6%) as black, and 31 patients (53.4%) as mixed race. Poor socioeconomic status was declared by 51 patients (87.9%). Symptoms related to the upper GI tract were present in 39 subjects (67.2%), pyrosis in 21 patients (36.2%), postprandial distress in 18 patients (31%), and epigastric pain in 17 patients (29.3%).

### 3.2. Endoscopic Findings

EGD showed changes in 51 patients (87.9%). The most frequent macroscopic alterations were edema, erythema, and erosions. The stomach was the site with the highest frequency of lesions, found in 40 patients (69%), compared to the lesions of the esophagus in 30 patients (51.7%) and of the duodenum in 22 patients (37.9%).

In the esophagus, the most frequent diagnosis was erosive esophagitis, present in 25 patients (43.1%) and classified as grade A (Los Angeles) in 21 of 25 patients (84%), grade B in three of 25 patients (12%), and grade C in one of 25 patients (4%). Aphthous ulcers located in the distal third were identified in one patient (1.7%). 

In the stomach, gastritis was the most frequent finding, identified in 32 patients (55.2%), with the erosive type in 19 of 32 patients (59.4%), and the erythematous type in 13 of 32 patients (40.6%). The most frequent location of gastritis was the antrum, in 15 of 32 patients (46.9%), followed by concomitant involvement of the body and antrum in 12 patients (37.5%), and, less frequently, the involvement of the body in five patients (15.6%). The intensity was classified as mild in 26 patients (81.2%). Two active gastric ulcers (Sakita A2) and one duodenal ulcer (Sakita H1) were observed during EGD in one *H. pylori*-positive patient.

There was no association between the presence of erythema and gastric erosion and age, gender, time of diagnosis, smoking, alcohol intake, treatment, location, behavior, or activity of the disease.

The duodenum was the least affected site, presenting with a normal appearance in 36 patients (62.1%). Erosive duodenitis was the most frequent duodenal finding, present in eight patients (13.8%). Duodenal ulcers were identified in three patients (5.2%), two of whom were *H. pylori*-positive. Ulcer scars were identified in four patients (6.9%), two of whom were *H. pylori*-positive, and two of whom were *H. pylori*-negative. 

No strictures or fistulas were identified in the esophagus, stomach, or duodenum. 

The main endoscopic findings were similar between the patients in remission or active disease states. 

The frequency of erosive esophagitis, gastritis, and duodenitis was similar between patients with or without upper GI symptoms. The endoscopic findings in patients with CD according to the presence or absence of upper GI symptoms are listed in Table 2.

### 3.3. Histopathological Findings

Among the 58 subjects included, seven (12.1%) underwent esophageal biopsies due to erosion, aphthous ulcer associated with scars, prolongation of columnar epithelium, and whitish plaques suggestive of Candida esophagitis and eosinophilic esophagitis. Chronic esophagitis was identified in six patients (85.7%), spongiosis in three patients (42.9%), and basal hyperplasia in two patients (28.6%). Just one patient (14.3%) presented normal histopathology. 

**Figure 1:**
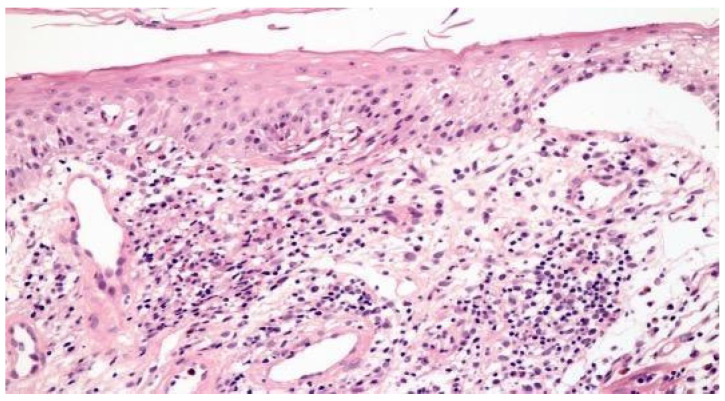
Focal esophagitis with spongiosis and subepithelial vesicle formation. Lamina propria with edema and mononuclear inflammatory infiltrate (H&E × 100).

Figure 1 illustrates the histopathological findings in one patient with aphthous ulcer in distal esophagus. 

**Figure 2:**
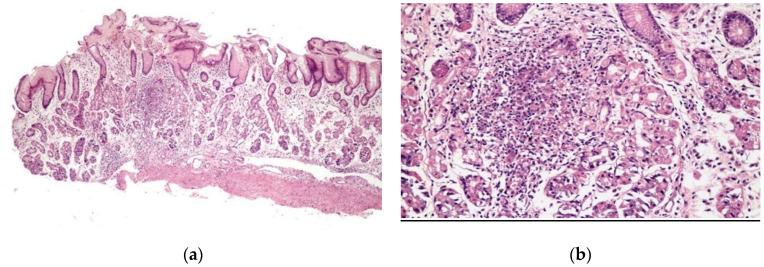
Focal enhanced gastritis: mononuclear inflammatory infiltrate associated with focal destruction of glands. Lesion intensity contrasts with slight inflammatory infiltrate in the remaining lamina propria. (**a**) H&E × 40; and (**b**) H&E × 100.

Chronic gastritis was identified in the body in 52 patients (89.7%) and in the antrum in 51 patients (87.9%), with a predominance of mononuclear cells in 37 patients (63.8%) and discrete infiltrate of the lamina propria in 35 patients (60.3%). Histopathological alterations were present in 18 of 19 patients (94.7%) of the patients with erosive gastritis, and in 13 of 13 patients (100%) with erythematous gastritis. Focally enhanced gastritis (FEG) was identified in four patients (6.9%), all of whom were *H. pylori-*negative (Figure 2), two with erythematous gastritis and two with erosive gastritis. Epithelioid granuloma was not found.

In the duodenum, the main findings were duodenitis in four patients (6.9%), with lymphocytic infiltrate in three patients (5.2%), mild activity in three patients (5.2%), and moderate activity in one patient (1.7%); intraepithelial lymphocytosis in one patient (1.7%), gastric metaplasia in one patient (1.7%), focal fibrosis in one patient (1.7%), and hyperplasia of Brunner glands in four patients (6.9%). Duodenal biopsies were normal in 47 patients (81%).

### 3.4. Comparison between H. Pylori-Positive and H.-Pylori-Negative Patients

The clinical characteristics were similar between the *H. pylori-*positive and *H. pylori*-negative patients.

The main endoscopic and histological findings among the patients positive and negative for *H. pylori* are shown in Table 3 and Table 4, respectively.

Endoscopic findings were compared between the *H. pylori*-positive and *H. pylori-*negative patients, with a higher frequency of antral involvement observed in the *H. pylori*-negative patients and a higher frequency of pangastritis and a greater intensity of gastritis observed in the *H. pylori*-positive patients, with no statistically significant difference (*p* > 0.05).

The presence of *H. pylori* was associated with increased inflammatory activity in the body and antrum, with a predominance of mononuclear and polymorphonuclear cells and permeation of inflammatory cells around the glands, whereas in the *H. pylori*-negative group, the inflammatory activity was discrete with a predominance of mononuclear cells (*p* < 0.05).

## 4. Discussion

The presence of upper GI involvement in patients with CD is variable and has been poorly described. Existing studies are heterogeneous regarding the description of upper GI symptoms, the number and location of biopsies, and specific protocols used for histopathological analysis. This is the first study from Latin America to evaluate the clinical, endoscopic, and histopathological aspects of the upper GI tract, exclusively in patients with CD, in a region of high endemicity for *H. pylori* infection.

Although we observed a high frequency of milder erosive esophagitis, this finding is considered nonspecific [19]. We identified one patient with an aphthous ulcer located in the distal third with mononuclear inflammatory infiltrate at the time of diagnosis, findings that may suggest an association with CD. Some studies have reported that superficial ulcers and erosions were the most frequent endoscopic findings, with an increase in chronic inflammatory infiltrate [20,21,22,23,24,25]. Therefore, in a patient with CD and esophageal lesions, even if superficial, it is important to perform biopsies, to confirm the involvement and to rule out other conditions such as esophageal reflux disease and infection.

In the stomach and in the duodenum, the most frequent endoscopic findings were edema, erythema, and erosions. Our data are in line with the literature in which gastric nonspecific inflammation was the most common finding described [10,26]. Erosions located in the gastric antrum are described at frequencies ranging from 24 to 73% and are difficult to distinguish from erosive gastritis due to other etiologies [16]. Duodenal lesions are reported at a frequency of 21 to 32.1% and include erosions, ulcers, and a notch-like appearance [23]. Sakuraba et al. [25] and Horje et al. [5] considered the presence of erosions to be a criterion for GI tract involvement when they were associated with suggestive histopathological findings such as FEG, epithelioid granuloma, and crypt distortion [18]. The finding of gastroduodenal erosions alone in patients negative for *H. pylori* does not meet the criteria necessary to define the involvement of these segments, and the correlation between endoscopic and histopathological findings is important [27].

In the histopathological analysis, our results demonstrated that all *H.-pylori*-negative patients presented with chronic inactive gastritis, characterized by the presence of lymphocytes with no evidence of granulocytes. Although this type of gastritis has been described in patients with CD, chronic active gastritis is more often related. Sonnenberg et al. [28] demonstrated that both *H. pylori-*negative chronic active gastritis and *H. pylori-*negative chronic inactive gastritis were more frequent in patients with IBD compared to controls. The finding of chronic inactive gastritis in our sample may be associated with previous treatment for *H. pylori* or previous use of PPI, since there may be persistence of the lymphocytic infiltrate in these situations, with no evidence of neutrophils and a higher frequency of *H. pylori-*negative chronic inactive gastritis [29,30,31].

Sonnenberg et al. [28] demonstrated that FEG had an increased prevalence in patients with IBD when compared to healthy controls. The prevalence of FEG was 43% to 71.4%, according to some authors [5,30,32]. Parente et al. [32] related a prevalence of 12% in patients with UC and 19% in controls, demonstrating that although this is a frequent finding, it is not specific to CD and can be found in other clinical conditions. The identification of FEG requires multiple gastric biopsies at different sites, which may make its identification difficult in clinical practice [28]. The few cases observed in our sample, all of them without *H. pylori* infection, may be explained by the limited number of biopsies performed. Since *H. pylori* may be associated with FEG, it is necessary to rule out this infection before associating this finding with CD.

Giemsa staining was positive for *H. pylori* in 32.8% of our patients, which was lower than expected in a population with a high prevalence of this infection. In Brazil, the prevalence is considered high, estimated to be present in 71.2% of the population [33]. The lower prevalence of this infection in patients with CD could be justified by the frequent use of some medications, such as antibiotics and sulfasalazine, or by immunological mechanisms unknown [30]. Two meta-analyses, performed by Luther et al. [11] and Wu X et al. [34], demonstrated a prevalence of *H. pylori* infection of 27.1% and 24.9% in patients with IBD, compared with 40.9% and 48.3% in the control groups without IBD, respectively. The lower prevalence identified in our study may also be associated with prior eradication status, recent use of antibiotics, or the use of only one method to detect the bacteria.

In our study, gastroduodenal granulomas were not identified. The prevalence of granulomas is variable and their identification depends on whether the material is the result of a surgical specimen or biopsy fragments, the biopsied site, and the number of fragments removed [23,35]. The finding of granulomas is less frequent in patients taking anti-TNF, since granuloma formation is dependent on inflammatory cytokines and alpha-TNF [4]. Some studies have demonstrated a correlation between a younger age, short history of disease, and the presence of granulomas [36]. The time of diagnosis, the age range of the patients included, the use of anti-TNF by almost one-third of the patients, and the conventional histopathological analysis may have influenced the lack of detection of granulomas in our sample.

Recent observational studies have been shown a higher frequency of involvement of the esophagus, stomach, and duodenum, with a prevalence between 16% to 41%. This involvement was more frequently identified when the endoscopy was performed at the time of diagnosis, independent of the presence of gastrointestinal symptoms [4,5]. Additional studies are needed to confirm the benefit of EGD to evaluate the extent of disease in adult asymptomatic patients at the time of diagnosis. Despite this greater frequency of endoscopic findings when endoscopy is performed at the moment of diagnosis, many findings are nonspecific with uncertain relevance.

The shortcomings of our study are the small number of patients, the single-center-based design, the absence of a control group without CD for comparison in our results, and the use of only one method for the detection of *H. pylori.* The majority of patients were in treatment with immunosuppressive or biological therapies at the time of endoscopy, which may have led to underestimation of the presence of more specific findings, such as epithelioid granuloma. Other relevant aspects not evaluated were the recent use of antibiotics, prior eradication status of *H. pylori*, and identification of chronic users of PPI.

Studies evaluating the frequency of endoscopic and histopathological findings in the upper GI tract in patients with CD are scarce; some have been retrospective and only one was designed to evaluate the prevalence at the time of diagnosis in all adult patients included. Another relevant aspect is that our study used the same endoscopist and pathologist, both blinded to the clinical presentation, reducing the observation bias. Furthermore, the pathologist was blinded to the endoscopic aspects of the study. The absence of more specific findings such as epithelioid granuloma, and the few cases of FEG detected in this sample, may reflect the difficulty of diagnosing upper GI tract involvement in clinical practice via the histopathological study of conventional biopsies from patients under treatment.

## 5. Conclusions

Nonspecific endoscopic and histopathological findings in the esophagus, stomach and duodenum were frequent in patients with CD. No association was observed between symptoms and endoscopic findings. The most frequent endoscopic and histopathological findings in *H. pylori-*negative patients were antral erosive gastritis and inactive chronic gastritis, respectively. FEG was uncommon and reported only in *H. pylori* negative patients. The presence of *H. pylori* was associated with increased inflammatory activity in the body and antrum, with a predominance of mononuclear and polymorphonuclear cells. The time from diagnosis, patient age, and drug therapy in use may have influenced the nondetection of epithelioid granuloma. New studies evaluating UGI involvement at the time of diagnosis, including protocols for endoscopic biopsy and histopathological analysis aiming to improve the detection of more specific findings, could be crucial to defining the real prevalence of CD in the upper GI tract and finally deciding on the need or not to perform EGD in newly diagnosed adult patients with CD.

## Figures and Tables

**Table 1:** Clinical and Demographic Characteristics of Patients with CD from Salvador/BA.

Characteristics	(*n* = 58)
Female, *n* (%)	30 (51.7)
Age (y), mean ± SD	42.1 ± 12.79
Age at diagnosis (y), mean ± SD	36.6 ± 12.26
Duration of CD (m), median (range)	48 (1-312)
Montreal disease age at diagnosis, *n* (%)	–
A1 (16 y or younger)	–
A2 (17–40 y)	40 (69)
A3 (over 40 y)	18 (31)
Montreal disease location, *n* (%)	–
L1 (terminal ileum)	9 (15.5)
L2 (colonic)	23 (39.7)
L3 (ileocolonic)	26 (44.8)
L4 (upper gastrointestinal tract)	3 (5.2)
Montreal disease behavior, *n* (%)	–
B1 (nonstricturing, nonpenetrating)	36 (62.1)
B2 (stricturing)	12 (20.7)
B3 (penetrating)	10 (17.2)
Perianal	23 (39.7)
Perianal fistula, *n* (%)	17 (29.3)
HBI, *n* (%)	–
Remission	35 (60.3)
Activity	23 (39.7)
UGI symptoms, *n* (%)	39 (67.2)
CRP elevated, *n* (%)	16 (27.6)
Anemia, *n* (%)	15 (25.9)
Smoking status, *n* (%)	–
Active	6 (10.3)
Absent	52 (89.7)
Alcohol status, *n* (%)	–
Active	8 (13.8)
Absent	50 (86.2)
Treatment, *n* (%)	55 (94.8)
Sulfasalazine	8 (13.8)
Topic mesalamine	8 (13.8)
Oral mesalamine	6 (10.3)
Azathioprine	40 (69)
Methotrexate	1 (1.7)
Adalimumab	6 (10.3)
Infliximab	12 (20.7)
Steroid therapy	6 (10.3)
Surgery, *n* (%)	13 (22.4)

Values are presented as mean ± SD, median (range), or number (%); y: years; m: months; CD: Crohn’s disease; HBI: Harvey Bradshaw Index; and CRP: C-reactive protein.

**Table 2:** Endoscopic Findings in Patients with CD According to the Presence of Upper GI Symptoms.

Endoscopic Findings	Present Upper GI Symptoms	Absent Upper GI Symptoms	*p* Value
	(*n* = 39)	(*n* = 19)	
Normal endoscopy	5 (12.8)	2 (10.5)	1.00 ^b^
Altered endoscopy	34 (87.2)	17 (89.5)	–
Esophagus	–	–	–
Erosive esophagitis (Los Angeles)	17 (43.6)	8 (42.1)	0.92 ^a^
A	14 (35.9)	7 (36.8)	–
B	2 (5.1)	1 (5.3)	–
C	1 (2.6)	–	–
Normal	18 (46.2)	10 (52.6)	0.64 ^a^
Stomach	–	–	–
Gastritis	22 (56.4)	10 (52.6)	0.14 ^b^
Erythematous	11 (50)	2 (20)	–
Erosive gastritis	11 (50)	8 (80)	–
Gastritis location	–	–	–
Body	4 (18.2)	1 (10)	0.27 ^b^
Antrum	8 (36.4)	7 (70)	0.22 ^b^
Pangastritis	10 (45.4)	2 (20)	–
Gastritis intensity	–	–	0.14 ^b^
Mild	16 (72.7)	10 (100)	–
Moderate to severe	6 (27.3)	–	–
Nodularity	7 (17.9)	3 (15.8)	1.00 ^b^
Ulcers (Sakita classification)	–	–	–
A2	1 (100)	–	–
Normal	13 (33.3)	5 (26.3)	0.59 ^a^
Duodenum	–	–	–
Duodenitis	–	–	1.00 ^b^
Erythematous	4 (10.3)	1 (5.3)	–
Erosive	5 (12.8)	3 (15.8)	–
Ulcer (Sakita classification)	–	–	0.43 ^a^
A2	3 (7.7)	–	–
S2	2 (5.1)	2 (10.5)	–
Normal	23 (59)	13 (68.4)	0.47 ^a^

Values are presented as numbers (%); ^a^ Pearson chi-squared test; ^b^ Fisher’s exact test; and GI: gastrointestinal.

**Table 3:** Comparison of Endoscopic Findings According with the Presence of *H. pylori* in Patients with CD.

Endoscopic Findings	Positive *H. Pylori* (*n* = 19)	Negative *H. Pylori* (*n* = 39)	*p* Value
Normal endoscopy	1 (5.3)	6 (15.4)	0.41 ^b^
Altered endoscopy	18 (94.7)	33 (84.6)	–
Esophagus	–	–	–
Erosive esophagitis	8 (42.1)	17 (43.6)	0.91 ^a^
Normal	9 (47.7)	19 (48.7)	0.92 ^a^
Stomach	–	–	–
Gastritis	11 (57.9)	21 (53.8)	0.77 ^a^
Erythematous	5 (45.5)	8 (38.1)	0.72 ^b^
Erosive	6 (54.5)	13 (61.9)	–
Gastritis location	–	–	0.13 ^b^
Body	2 (18.2)	3 (14.3)	–
Antrum	3 (27.3)	12 (57.1)	–
Pangastritis	6 (54.5)	6 (28.6)	–
Gastritis intensity	–	–	0.15 ^b^
Mild	7 (63.6)	19 (90.5)	–
Moderate to severe	4 (36.4)	2 (9.5)	–
Nodularity	5 (26.3)	5 (12.8)	0.27 ^b^
Ulcers (Sakita classification)	–	–	–
A2	1 (100)	–	–
Normal	4 (21.1)	14 (35.9)	0.25 ^a^
Duodenum	–	–	–
Duodenitis	–	–	–
Erythematous	2 (10.5)	3 (7.7)	1.00 ^b^
Erosive	2 (10.5)	6 (15.4)	
Ulcers (Sakita classification)	–	–	1.00 ^b^
A2	2 (10.5)	1 (2.6)	–
S2	2 (10.5)	2 (5.1)	–
Pseudopolyp	–	1	–
Flat lesions	1 (5.3)	2 (5.1)	–
Normal	12 (63.2)	24 (61.5)	0.91 ^a^

Values are presented as numbers (%); ^a^ Pearson chi-square test; ^b^ Fisher’s exact test; CD: Crohn’s disease; and *H. pylori: Helicobacter pylori.*

**Table 4:** Comparison of Histopathological Findings According with the Presence of *H.-pylori* in Patients with CD.

Histopathological Findings	Positive *H. pylori* (*n* = 19)	Negative *H. pylori* (*n* = 39)	*p* Value
Normal	0	3 (7.7)	0.54 ^b^
Altered	19 (100)	36 (92.3)	–
Stomach	–	–	–
Antrum chronic gastritis	19 (100)	32 (82.1%)	0.08 ^b^
Activity	–	–	<0.001 ^a^
Present	18 (94.7)	6 (15.4)	–
Absent	1 (5.3)	33 (84.6)	–
Body chronic gastritis	19 (100)	33 (84.6)	0.16 ^b^
Activity	–	–	<0.001 ^a^
Present	18 (94.7)	4 (10.3)	–
Absent	1 (5.3)	35 (89.7)	–
Inflammatory cells	–	–	–
Mononuclear	2 (10.5)	35 (89.7)	<0.001 ^a^
Mononuclear and granulocytes	17 (89.5)	–	–
Absent	0	4 (10.3)	–
Lamina propria infiltrate	–	–	0.29 ^b^
Present	19 (100)	35 (89.8)	–
Absent	–	4 (10.3)	–
Permeation of glands by inflammatory cells	14 (73.7)	–	<0.001 ^a^
Foveolar hyperplasia	–	3 (7.7)	–
Intestinal metaplasia	1 (5.3)	–	–
Atrophy	1 (5.3)	–	–
Focally enhanced gastritis	–	4 (10.3)	–
Normal	–	5 (12.8)	0.16 ^b^
Duodenum	–	–	–
Intraepithelial lymphocytosis	–	1 (2.6)	–
Gastric metaplasia	1 (5.3)	–	–
Duodenitis	1 (5.3)	3 (7.7)	–
Duodenitis (activity)	–	–	1.00 ^b^
Present	1 (5.3)	3 (7.8)	–
Absent	18 (94.7)	36 (92.3)	–
Duodenitis (inflammatory cells)	–	–	–
Mononuclear	1 (5.3)	2 (5.1)	–
Mononuclear and granulocytes	–	1 (2.6)	–
Absent	18 (94.7)	36 (92.3)	–
Normal	16 (84.2)	31 (79.5)	1.00 ^b^

Values are presented as numbers (%); ^a^ Chi-square test; ^b^ Fisher’s exact test; and *H. pylori: Helicobacter pylori.*

## Data Availability

The data used to support the findings of this study are available from the corresponding author upon request.
